# Neonatal mortality and morbidity among infants between 24 to 31 complete weeks: a multicenter survey in China from 2013 to 2014

**DOI:** 10.1186/s12887-016-0716-5

**Published:** 2016-11-03

**Authors:** XiangYong Kong, FengDan Xu, Rong Wu, Hui Wu, Rong Ju, XiaoLin Zhao, XiaoMei Tong, HongYan Lv, YanJie Ding, Fang Liu, Ping Xu, WeiPeng Liu, HongBin Cheng, TieQiang Chen, ShuJuan Zeng, WenZheng Jia, ZhanKui Li, HuiXian Qiu, Jin Wang, ZhiChun Feng

**Affiliations:** 1 Newborn Care Center, Bayi Children’s Hospital, the Army General Hospital, the People’s Liberation Army (PLA), 5 Nan Men Cang Hu Tong, Dongcheng District, Beijing, 100700 China; 2 Clinical Medical College, the Army General Hospital, Southern Medical University, Beijing, 100700 China; 3 Neonatal Medical Center, Huaian Maternity and Child Healthcare Hospital, Yangzhou University Medical School, Huaian, 223002 China; 4 Department of Pediatrics, the First Hospital of Jilin University, Changchun, 130021 China; 5 Newborn Care Center, Chengdu Women’s and Children’s Central Hospital, Chengdu, 610000 China; 6 Department of Neonatology, North-west Women’s and Children’s Hospital, Xi’an, 710003 China; 7 Department of Neonatology, The Third Hospital of Beijing University, Beijing, 100191 China; 8 Department of Neonatology, Handan Women’s and Children’s Hospital, Handan, 056001 China; 9 Department of Pediatrics, Yantai Yuhuangding Hospital, Yantai, 264000 China; 10 Department of Neonatology, Bethune International Peace Hospital, Shijiazhuang, 130100 China; 11 Department of Pediatrics, Liaocheng People’s Hospital, Liaocheng, 252004 China; 12 Department of Pediatrics, Navy General Hospital, Beijing, 100048 China; 13 Department of Pediatrics, Huangshi Women’s and Children’s Hospital, Huangshi, 435003 China; 14 Department of Neonatology, Changsha Hospital for Maternal and Child Health Care, Changsha, 410007 China; 15 Department of Pediatrics, Longgang Central Hospital of Shenzhen, Shenzhen, 518116 China; 16 Department of Neonatology, 302 Hospital of P.L.A, Beijing, 100039 China

**Keywords:** Preterm infants, Mortality, Morbidity, Outcome

## Abstract

**Background:**

The outcome of preterm infants has been varied in different hospitals and regions in developing countries. Regular clinical monitor are needed to know the effects of health care. This study aimed to describe the survival and morbidity rates of extreme to very preterm infants in 15 neonatal-intensive care hospitals in China.

**Methods:**

Data were collected from January 1, 2013 to December 31, 2014 for preterm neonates with gestational age (GA) between 24 and 31 complete weeks born in hospitals from our collaborative study group. The primary outcomes were survival and major morbidities prior to hospital discharge. Major morbidities included bronchopulmonary dysplasia (BPD), intraventricular hemorrhage (IVH), necrotizing enterocolitis (NEC), retinopathy of prematurity (ROP), patent ductus arteriosus (PDA) and sepsis. Mutivariate logistic regression was used to analyze the risk factor influencing on the outcomes.

**Results:**

The preterm birth rate was 9.9 % (13 701/138 240). The proportion of extreme to very preterm infants was 1.1 % and 11.8 % respectively. The survival rate prior to discharge was increased with increasing GA (0, 24 weeks; 28 %, 25 weeks; 84.8 %, 26 weeks; 83.5 %, 27 weeks; 87.4 %, 28 weeks; 90.7 %, 29 weeks; 93.9 %, 30 weeks; 96 %, 31 weeks). Rate of survival and without severe morbidity according to GA were 0 at 24 weeks, 8 % at 25 weeks, 60.6 % at 26 weeks; 53.2 % at 27 weeks; 62.3 % at 28 weeks; 67.9 % at 29 weeks; 79.1 % at 30 weeks, 85.8 % at 31 weeks respectively. Rate of antenatal steroid use was 56 %. The antenatal steroid use was lower in GA < 28 weeks infants than that in GA between 28 and 32 weeks (28–44.3 % vs 49.7–60.1 %, *P* < 0.05). Infants at the lowest GAs had a highest incidence of morbidities. Overall, 58.5 % had respiratory distress syndrome, 12.5 % bronchopulmonary dysplasia, 3.9 % necrotizing enterocolitis, 15.4 % intraventricular hemorrhage, 5.4 % retinopathy of prematurity, 28.4 % patent ductus arteriosus, and 9.7 % sepsis. Mortality and morbidity were influenced by gestational age (OR = 0.891, 95 % CI: 0.796–0.999, *p* = 0.0047 and OR = 0.666, 95 % CI: 0.645–0.688, *p* = 0.000 respectively), birth weight (OR = 0.520, 95 % CI: 0.420–0.643, *p* = 0.000 and OR = 0.921, 95 % CI: 0.851–0.997, *p* = 0.041 respectively), SGA (OR = 1.861, 95 % CI: 1.148–3.017, *p* = 0.012 and OR = 1.511, 95 % CI: 1.300–1.755, *p* = 0.000 respectively), Apgar score <7 at 5 min (OR = 1.947, 95 % CI: 1.269–2.987, *p* = 0.002 and OR = 2.262, 95 % CI: 1.950–2.624, *p* = 0.000 respectively). The survival rate was increased with more prenatal steroid use (OR = 1.615, 95 % CI: 1.233–1.901, *p* = 0.033).

**Conclusion:**

Although most of the preterm infants with GAs ≥26 weeks survived, a high complication in survivors still can be observed. Rate of survival of GAs less than 26 weeks was still low, and quality improvement methods should be used to look into increasing the use of antenatal steroids in the very preterm births.

## Background

In 2010, the national preterm birth rate was estimated at 14.9 million infants or more than one in ten of all infants in 184 countries [1]. More than a million of those infants died mainly because of severe complications associated with premature birth. Prematurity was the leading cause of death in the first month of life and was a factor in greater than 75 % of early deaths in the neonatal period [1]. Across all regions, although infants born at less than 32 weeks account for about 16 % of all preterm births, mortality and morbidity were the highest among these infants [2]. Even though there have been ongoing advances in neonatal intensive care and a significant decline in neonatal mortality in the past several decades, there is still much room for improvement in morbidity and mortality among very preterm infants (VPI: gestational age <32 weeks) and extremely preterm infants (EPI: gestational age <28 weeks) [3–5]. In order to make the best solution for the prevention of morbidity and mortality in VPI and EPI, it is essential to continue monitoring the outcomes among these high-risk infants.

Although China is one of the most populous countries in the world, the outcomes of newborn infants and the associated risk factors are only estimated by vital statistics. It may address public health concerns with regards to overall mortality and related causes, but does not answer questions pertaining to specific perinatal and neonatal care, gestation specific mortalities and morbidities, risk factors, as well as resuscitation and postnatal care of these high risk newborns [6]. In China, only a few publications analyzed the short-term outcome of preterm infants on a nationwide basis over more than ten years. We previously published a single-center age-specific outcome study of preterm infants less than 32 weeks gestation born between 2010 and 2012 [7]. However, more recently, several national neonatal networks have established and compared their data to assess the differences in outcomes, and to identify the progress in the whole areas. On this ground, the aim of our current study is to evaluate the hospital outcomes of the live born preterm infants less than 32 weeks gestation at birth admitted to any of the 15 hospitals in China from 2013 to 2014. And to format gestational age-specific hospital survival rates and major morbidity rates taking into account common perinatal risk factors that were known to affect the outcomes of preterm infants. We also compared the current outcomes for VPI with outcomes from other countries.

## Methods

### Participating centers

We established a collaborative study group including nine general hospitals and six maternal and children’s hospitals from major cities of 11 provinces in China. All the participating hospitals are tertiary neonatal care centers and have their own maternity wards in local region, which are representatives of medical units providing newborn intensive care in their respective areas. Nine neonatal intensive care units (NICU) of all the participating hospitals provide regional perinatal and neonatal retrieval services. In this prospective study design, our study was approved by the Institutional Ethics Committee of the Army General Hospital, the People’s Liberation Army (PLA). The approval of data collection was also obtained at each of the collaborating hospitals. This study was also conducted as part of the Neonatal Research Network of the Chinese Neonatologist sub-association of the Chinese Medical Doctor Association. Coordination for this study was based at the Army General Hospital of PLA.

### Study population and clinical outcomes

Infants born alive at hospitals of our collaborative study group in 2013–2014 with GAs of 24^+0^ to 31^+6^ weeks were studied, excluding those with congenital anomalies. Outborn infants were also excluded in this investigation. Researchers collected maternal pregnancy/delivery data soon after birth and infant data from birth to death or discharge. To minimize bias among centers and investigators, we provided comprehensive and systematic training for the staffs committed to the survey. Data collection by research staff at each of the collaborating hospitals was supervised by the NICU director, who was responsible for the quality assurance. The quality control was mainly focused on completeness and accuracy of the contents in records by each unit investigators, along with visiting, telephone or email communication for verification and correction of the data. Records were also subjected to collaborative center to check for accuracy and completeness and returned for correction if needed.

GA was determined by the most experienced obstetric physicians estimated by the date of last menstrual period. Preterm infants was defined as a GA <37 completed weeks. Extremely preterm infant (EPI) was defined as GA < 28 weeks and very preterm infants (VPI) was defined as GA < 32 weeks. Small for gestational age (SGA) infant was defined as birth weight of <10th percentile for gender and GA, by using growth charts published by Fenton and Kim [8]. Birth weight was recorded within 1 h of birth. Antenatal care was defined as receiving an obstetrician at least one occasion during pregnancy.

Morbidities were defined according to the earlier publications [3, 9–11], including: (1) respiratory distress syndrome (RDS): (i)clinical evidence of respiratory difficulties (tachypnea, retraction, grunting and cyanosis), (ii)radiographic appearance of RDS (low volume lungs with a diffuse reticulogranular pattern and air bronchograms). (2) bronchopulmonary dysplasia (BPD): Infants were considered as having BPD if they required mechanical ventilation or supplemental oxygen 28 days after birth. Moderate to severe BPD were defined as infants who still received supplemental oxygen at 36 weeks’ postmenstrual age (PMA) or at discharge. (3) intraventricular hemorrhage (IVH): IVH was diagnosed within 28 days of birth by using real-time portable cranial ultrasound. Severe IVH was defined as Grades III and IV according to the Papile grading system based on head ultrasound. (4) necrotizing enterocolitis (NEC): The diagnosis of NEC required ≥1abdominal sign (eg, bilious gastric aspirate or vomitting, abdominal distention or tenderness, gross or occult blood in the stool) and ≥1 radiographic finding (eg, pneumatosis intestinalis, hepatobiliary gas, a fixed position loop on serial studies). High grade NEC was ≥ grade II according to modified Bell’s criteria. (5) retinopathy of prematurity (ROP): The diagnosis and staging of ROP were based on retinal examination before discharge with severe ROP defined as stages 3 to 5. (6) Sepsis, including positive blood culture and clinical diagnosis.

Mortality was defined as death in the delivery room or in NICU before discharge. Infants were considered as having a major neonatal morbidity if they had at least one of the following complications before discharge: moderate to severe BPD, severe IVH, high grade NEC, severe ROP and sepsis. Combined outcome refers to infants who died or survived with a major neonatal morbidity. Infants who died in delivery room were excluded from analyses focus on morbidity diagnosed later after birth.

### Statistical analysis

All statistical analyses were conducted by using SPSS 16.0 software (SPSS, Chicago, IL). Continuous variables are described as means and SD. Categorical variables are presented as rates and odds ratio with 95 % confidence intervals (CI). Comparison between continuous variables was conducted by using a One-way ANOVA. Univariate analyses on categorical variables were made by using a 2-tailed Pearson *χ* ^2^ or Fisher’s exact test wherever appropriate.

Multivariate analyses were performed separately by using logistic regression to analyze the risk factors of mortality and morbidity in preterm infants. GA, gender, maternal perinatal complications such as: Gestational diabetes, Placenta previa, placental abruption, hypertensive disorder complicating pregnancy (HDCP) and Premature rupture of membrane (PROM), multiple birth, delivery mode, Small for GA, Apgar score and antenatal steroid use were included in the analysis. *P* < 0.05 was considered statistically significant.

## Results

### Incidence of preterm birth

During a two-year period, there were 138 240 alive born infants including 13 701 preterm infants from the 15 participating hospitals. The incidence of preterm birth rate was 9.9 %. Of these, there were totally 1 760 infants born before 32 weeks (including 148 and 1 612 EPI and VPI, corresponding to 1.1 % and 11.8 % of all preterm infants respectively). The number of preterm infants increased with increasing GA. See Table 1.

**Table 1:** Neonatal characteristics of the study infants by gestational age

Gestational age	24 weeks	25 weeks	26 weeks	27 weeks	28 weeks	29 weeks	30 weeks	31 weeks	Total
Numbers (%)	11 (0.6)	25 (1.4)	33 (1.9)	79 (4.5)	199 (11.3)	321 (18.2)	444 (25.2)	648 (36.8)	1760 (100)
Males (%)	4 (36.4)	15 (60.0)	22 (66.7)	47 (59.5)	122 (61.3)	173 (53.9)	267 (60.1)	351 (54.2)	1001 (56.9)
Birth weight (g)	682.7 ± 99.6	799.6 ± 306.5	1022.8 ± 453.7	1004.2 ± 157.2	1179.7 ± 194.8	1306.8 ± 259.6	1639.9 ± 325.0	1828.5 ± 357.6	1463.0 ± 605.6
Multiple birth (%)	3 (27.3)	12 (48.0)	7 (21.2)	34 (43.0)	56 (28.1)	76 (23.7)	124 (27.9)	202 (31.2)	514 (29.2)
Caesarean section (%)	1 (9.1)	3 (12.0)	6 (18.2)	14 (17.7)	64 (30.7)	111 (34.6)	185 (41.7)	326 (50.3)	710 (40.3)
SGA (%)	0 (0)	4 (16.0)	1 (3.0)	2 (2.5)	10 (5.0)	18 (5.6)	37 (8.3)	44 (6.8)	116 (6.6)
5 min Apgar score <7 (%)	7 (63.6)	5 (20.0)	12 (36.4)	20 (25.3)	33 (16.6)	25 (7.8)	36 (8.1)	21 (3.2)	159 (9.0)
Antenatal care (%)	9 (81.8)	20 (80.0 %)	29 (87.9)	70 (88.6)	174 (87.4)	286 (89.1)	401 (90.3)	589 (90.9)	1578 (89.7)
Antenatal steroid use (%)	0 (0)	7 (28.0)	11 (33.3)	35 (44.3)	99 (49.7)	186 (57.9)	262 (59.0)	386 (59.6)	986 (56.0)
Complete antenatal steroid use (%)	0 (0)	3 (12.0)	5 (15.2)	17 (21.5)	48 (24.1)	115 (35.8)	159 (35.8)	221 (34.1)	568 (32.3)

### Characteristics of study infant

Overall, there were 1 001(56.9 %) male infants, 514(29.2 %) multiple births, and 116(6.6 %) small for gestational age infants. Infants with Apgar scores of <7 at 5 min represented 9.0 % of all EPI and VPI, and the incidence was high in lower GA infants. Caesarean sections were done in 710 (40.3 %) infants and the rate was increased with increased GA, with 9.1 % at 24 weeks and 50.3 % at 31 weeks (OR 1.168; 95 % confidence interval (CI) 1.149–1.187; *p* = 0.000). The majority (89.7 %) of women received antenatal care. Rate of antenatal steroid use increased with advancing GA, from 0 at 24 weeks to 44.3 % at 27 weeks and 49.7 % to 59.6 % at 28 to 31 weeks. The rate of complete antenatal steroid use was 32.3 % and also increased with GA. (see Table 1).

### Hospital survival rate

54/1 760 (3.1 %) live born infants died during the first week after birth, ranging from 54.5 % at 24 weeks, 3.5 % at 28 weeks to 0.3 % at 31 weeks of gestation. In 22.2 % of the early deaths, a discontinued treatment policy was made according to parents’ opinion before birth: 45.5 % at 24 weeks of gestation, 15.2 % at 25 weeks, 3.0 % at 26 weeks of gestation. In total, there was no infant survived at 24 weeks gestation. Rates of survival before discharge increased with the increase of GA, from 28 % at 25 weeks, 84.8 % at 26 weeks, 83.5 % at 27 weeks, 87.4 % at 28 weeks, 90.7 % at 29 weeks, 93.9 % at 30 weeks and to 96.0 % at 28 weeks (91.2 % overall). There was a large increase in survival of greater than 50 % from 25 week to 26 gestational weeks. The survival rate was over 90 % in 29 weeks, 30 weeks and 31 weeks of gestation. (Table 2).

**Table 2:** Survive and morbidity rate according to GA for infants <32 weeks from 2013 to 2014

Gestational age	24 weeks	25 weeks	26 weeks	27 weeks	28 weeks	29 weeks	30 week	31 week	Total
Numbers (%)	11	25	33	79	199	321	444	648	1760
Died within 7 days (%)	6 (54.5)	13 (52.0)	3 (9.1)	8 (10.1)	7 (3.5)	9 (2.8)	6 (1.4)	2 (0.3)	54 (3.1)
Died before discharge (%)	5 (45.5)	5 (20.0)	2 (6.1)	5 (6.3)	18 (9.1)	21 (6.5)	21 (4.7)	24 (3.7)	101 (5.7)
Survived to discharge (%)^a^	0 (0)	7 (28.0)	28 (84.8)	66 (83.5)	174 (87.4)	291 (90.7)	417 (93.9)	622 (96.0)	1605 (91.2)
Survived without morbidity (%)^b^	0 (0)	2 (8.0)	20 (60.6)	42 (53.2)	124 (62.3)	218 (67.9)	351 (79.1)	556 (85.8)	1313 (74.6)
Died or survived with at least one morbidity (%)	11 (100)	23 (92.0)	13 (39.4)	37 (46.8)	75 (37.7)	103 (32.1)	93 (20.9)	92 (14.2)	447 (25.4)
RDS (%)	10 (90.9)	22 (88.0)	29 (87.9)	69 (87.3)	167 (83.9)	223 (69.5)	234 (52.7)	276 (42.6)	1030 (58.5)
BPD (%)^c^	-	4 (57.1)	11 (39.3)	20 (30.3)	45 (25.9)	63 (21.6)	33 (7.9)	24 (3.9)	200 (12.5)
NEC (%)^d^	-	0 (0)	2 (6.7)	7 (9.9)	13 (6.8)	11 (3.5)	10 (2.3)	23 (3.6)	66 (3.9)
IVH (%)^d^	-	5 (41.7)	7 (23.3)	23 (32.4)	31 (16.1)	50 (16.0)	61 (13.9)	85 (13.2)	262 (15.4)
PDA (%)^d^	-	4 (33.3)	11 (36.7)	30 (42.3)	59 (30.7)	102 (32.7)	117 (26.7)	162 (25.1)	485 (28.4)
ROP (%)^c^	-	2 (28.6)	5 (17.9)	10 (15.2)	18 (10.3)	21 (7.2)	16 (3.8)	14 (2.3)	86 (5.4)
Sepsis (%)^a^	-	5 (20.0)	7 (21.2)	11 (13.9)	33 (16.6)	35 (10.9)	42 (9.5)	37 (5.7)	170 (9.7)

*RDS* respiratory distress syndrome, *BPD* bronchopulmonary dysplasia, including mild, moderate and severe BPD, *NEC* necrotizing enterocolitis, including stage I, II and III, *IVH* intraventricular hemorrhage, including grade I, II, III and IV, *ROP* retinopathy of prematurity; including stage I, II, III, IV and V. *PDA* Patent ductus arteriosus

^a^ Proportion among all infants including survivors

^b^Morbidity including moderate to severe BPD, severe IVH, high grade NEC, sepsis and ROP stage ≥3

^c^Proportion among infants who survived

^d^Proportion among infants who survived > 7 days

Mutivariate logistic regression analysis indicated that the mortality of GA < 32 weeks preterm infants was decreased with increasing gestational age (OR = 0.891, 95 % CI: 0.796–0.999, *p* = 0.0047), birth weight (OR = 0.520, 95 % CI: 0.420–0.643, *p* = 0.000), and more antenatal steroids use (OR = 1.615, 95 % CI: 1.233–1.901, *p* = 0.033). Infants with SGA and Apgar < 7 at 5 min died more often than infants with infants who have no these risk factors (OR = 1.861, 95 % CI: 1.148–3.017, *p* = 0.012; OR = 1.947, 95 % CI: 1.269–2.987, *p* = 0.002, respectively). (Table 3).

**Table 3:** Multivariate logistic regression analysis of risk factors for mortality and severe morbidity

Factors	Mortality					Morbidity				
	B value	Wald	OR	95 % CI	*P*	B value	Wald	OR	95 % CI	*P*
GA	−0.115	3.930	0.891	0.796–0.999	0.047	−0.406	624.097	0.666	0.645–0.688	0.000
Birth weight	−0.654	36.228	0.520	0.420–0.643	0.000	−0.082	4.160	0.921	0.851–0.997	0.041
male	0.165	1.655	1.180	0.917–1.518	0.198	0.211	22.444	1.235	1.132–1.347	0.000
Caesarean section	0.278	2.364	1.321	0.926–1.884	0.124	0.027	0.505	1.027	0.954–1.105	0.477
Antenatal steroid use	0.191	1.943	1.615	1.233–1.901	0.033	0.021	0.199	1.021	0.933–1.117	0.655
Gestational diabetes	−0.273	0.579	0.761	0.377–1.537	0.447	−0.099	1.521	0.906	0.775–1.060	0.218
SGA	0.621	6.349	1.861	1.148–3.017	0.012	0.413	29.072	1.511	1.300–1.755	0.000
PROM	−0.161	0.847	0.851	0.604–1.200	0.357	0.027	0.481	1.027	0.953–1.107	0.488
placental abruption	0.511	3.328	1.667	0.963–2.887	0.068	0.322	6.711	1.380	1.082–1.760	0.010
Placenta previa	−0.202	0.424	0.817	0.444–1.502	0.515	0.373	15.037	1.452	1.202–1.753	0.000
HDCP	0.141	0.421	1.151	0.753–1.760	0.517	0.190	7.358	1.209	1.054–1.387	0.007
Multiple birth	−0.017	0.009	0.983	0.698–1.384	0.923	0.150	9.502	1.162	1.056–1.278	0.002
Apgar < 7 at 5 min	0.666	9.321	1.947	1.269–2.987	0.002	0.816	116.087	2.262	1.950–2.624	0.000
Maternal fever before delivery	0.100	0.292	1.105	0.770–1.585	0.589	0.312	32.891	1.367	1.228–1.521	0.000

*GA* gestational age, *SGA* small for gestational age, *PROM* Premature rupture of membrane, *HDCP* hypertensive disorder complicating pregnancy

#### Major neonatal morbidity

The smaller of the GA, there was a greater risk of morbidities due to prematurity (Table 2). Overall, 58.5 % infants experienced RDS. The incidence of RDS was over 85 % among infants at 24–27 weeks. The risk of BPD was inversely proportional to the GA at birth. Because of the inclusion of infants with mild BPD, the incidence of BPD was 12.5 % in total with the severity-based definition of BPD. Overall, 15.4 % of cranial sonograms indicated IVH decreased with the increase of GA (41.7 % at 25 weeks and 13.2 % at 31 weeks). A highest incidence of sepsis was diagnosed at the lowest GA (20 % at 25 weeks and 5.7 % at 31 weeks). 3.9 % of infants developed NEC. PDA was diagnosed for 28.4 % of infants who survived >7 days. The average incidence of ROP was 5.4 % and the interval was from 28.6 % at 25 weeks to 2.3 % at 31 weeks. Rates of died or survived with at least one morbidity decreased with the increase of GA (100 % at 24 weeks to 92 % at 25 weeks, 39.4 % at 26 weeks, 46.8 % at 27 weeks, 37.7 % at 28 weeks, 32.1 % at 29 weeks, 20.9 % at 30 weeks, and 14.2 % at 31 weeks).

Mutivariate logistic regression analysis indicated that the morbidity of GA < 32 weeks preterm infants was decreased with increasing gestational age (OR = 0.666, 95 % CI: 0.645–0.688, *p* = 0.000) and birth weight (OR = 0.921, 95 % CI: 0.851–0.997, *p* = 0.041). SGA(OR = 1.511, 95 % CI: 1.300–1.755, *p* = 0.000), HDCP(OR = 1.209, 95 % CI: 1.054–1.387, *p* = 0.007), placental abruption(OR = 1.380, 95 % CI: 1.082–1.760, *p* = 0.010), placenta previa(OR = 1.452, 95 % CI: 1.202–1.753, *p* = 0.000), multiple birth(OR = 1.162, 95 % CI: 1.056–1.278, *p* = 0.002), Apgar < 7 at 5 min(OR = 2.262, 95 % CI: 1.950–2.624, *p* = 0.000) and maternal fever before delivery(OR = 1.367, 95 % CI: 1.228–1.521, *p* = 0.000) were associated with increased risk of morbidity.

## Discussion

The incidence of preterm birth and mortality is associated with the level of economic development, quality of medical technology and advance in perinatal care. Although preterm birth has decreased in some high-income countries, the worldwide rates have increased during the last decade [1, 2]. In 2010, the number of premature birth was about 14.9 million, account for 11.1 % of all live births worldwide, ranging from about 5 % in several European countries to 18 % in some African countries. In China, The rate of preterm infant had steadily increased from the early 2000s. In 2002–2003, the incidence of preterm infants was 7.8 % accounting for 19.7 % in hospitalization neonates [12]. The number was increased to 8.1 % in 2005 and to 8.81 % during 2010–2011 according to regional reports [13, 14]. In our data, rate of preterm birth was 9.9 %. Premature birth rate in China is now comparable to other developed countries and this is likely to increase over the years due to increase in maternal age and use of assisted conception, and also because many of the EPI and VPI might be reported stillbirth before. Improvement in maternal and newborn infant health care would have an impact on survival of the more premature infants. Although babies born at GA < 32 weeks accounted for 12.9 % of preterm births, they however accounted for >50 % of infant death and neurodevelopmental disabilities [3, 13, 14].

Although many previous reports used birth weight (BW) as the reference for morbidity and mortality, the current study assessed outcomes in accordance with GA. BW specific data may not be accurate because of the limited growth of more mature infants. Appreciation of GA-based outcomes is especially valuable for prenatal counseling and physician/family decision-making [11]. GA was determined mainly based on the first day of last menstrual period and not on early ultrasonic scan in this study because the accuracy of estimate by ultrasonography is decreased with increasing GA. The variation is up to 2 weeks at 28 weeks, and this may have an impact on the results of this study.

This study on outcome of 24 to 31 weeks premature infants in China showed a survival rate of 91.2 % (mortality rate was 8.8 %) among live born infants. 74.6 % of infants had discharged home without any severe morbidity during their hospital stay. The survival rate was slightly increased than that of reported in previous data in China, although the survival rate of EPI and VPI vary greatly. A study from Jiangsu province in China in 2010 had reported the survival rate of 25 weeks to 31 weeks was 0, 0, 58.3 %、66.7 %、60.3 %、82.9 % and 79.2 % respectively^6^. In Guangdong province of China, the survival rate of EPI was 20 % at 24 weeks、15.9 % at 25 week、27.2 % at 26 week and 48.4 % at 27 weeks. According to birth weight the survival rate of ELBW infants were also vary from 38.5 to 87 % in China [15]. In Canada, the Canada neonatal network (CNN) data showed significantly decrease in mortality rate of GA <29 weeks from 1996 to 1997 (17.2 %) to 2006–2007 (14.7 %) [16]. Isayama et al. had compared Neonatal Research Network of Japan (NRNJ) data with CNN data in 2006–2008, the mortality rate at GA < 25 weeks, 26–27 weeks, 28–29 weeks and 30–32 weeks were 27.1 % vs 52.3 %、9.6 % vs 17.9 %、4.1 % vs 7.3 % and 1.4 % vs 1.7 %. It indicated that the mortality rate was higher in Canada than that in Japan, especially in lowest GA [17]. New South Wales and the Australian Capital Territory Neonatal Intensive Care Units’ Data showed the survival rate of GA between 22 and 31 weeks was 26.8 %、59.1 %、75.6 %、85.1 %、91.1 %、95.6 %、97.2 %、97.8 % and 98.6 %. It indicated their survival rate was more than 95 % between 28 and 31 weeks [11]. Our data showed there was no survivor of infants for 24 weeks of GA and high rate in early death. Infants born at 25 weeks had more than three times the risk of death, compared with infants born at 26 weeks. The survival rate at 26 to 27 weeks and 28 to 31 weeks were steadily closed to developed countries. Furthermore, there is still controversy about whether to provide active obstetric care and to initiate neonatal intensive care to the EPI in China. There are no authoritative policies or guidelines for physicians to abide whether a 24 weeks infant should give intensive resuscitation or not. Mostly it was up to the parents’ opinion. It was common that some EPIs were stopped intensive care in the shortly after birth, in the early or late process of treatment due to shortage of money and poor prognosis. It means some of the infant may survive if treatment were to continue. On this ground, the estimate of survival rate will be slightly low. So there were unavoidable errors in some degree about the survival rate of these infants.

The incidence of hospital morbidity rate was still high among EPIs, and morbidities contribute to poor neurodevelopmental outcomes. Our data showed the combined outcome of death or ≥1 major neonatal morbidity in survivors was 100 % at 24 weeks and 92 % at 25 weeks, decreased to 39.4 % among 26 weeks and 14.2 % at 31 weeks. This is a big gap compared to data from other countries [18, 19]. In Japan, the NRNJ data showed the combined outcome of death and/or neurodevelopment impairment in survivors in 22 to 25 weeks was 80 %、63.7 %、38.9 % and 34.1 % respectively [18]. In the United States, the National Institute of Child Health and Human Development (NICHD) Neonatal Research Network showed that the composite outcome in 22 to 28 weeks was 100 % 22 weeks; 86 %–91 %, 23 weeks; 95 %–89 %, 24 weeks; 80 %–78 %, 25 weeks; 68 %–66 %, 26 weeks; 56 %–54 %, 27 weeks and 46 %–38 %, 28 weeks [19]. Most infants experienced a major complication during the first hospitalization, with the risk of morbidity being inversely related to GA at birth.

Many studies showed the major neonatal morbidities are predictive of long-term neurodevelopmental disorders such as cerebral palsy, cognitive impairment, hearing loss, and visual problem [16–22]. We can assess the risks for long-term neurodevelopmental disorders by using the results of Schmidt et al. [22], who reported that three neonatal morbidities including BPD, severe brain injury (grade III or IV IVH, PVL and porencephalic cysts), and severe ROP, strongly predict poor neurodevelopmental outcome (motor impairment, cognitive disorders, behavior problems, poor general health, hearing loss, and visual problem) at 5 years for very low birth infants. The incidence of BPD, serious brain injury, and severe ROP were account for 43 %, 13 %, and 6 % in all infants, respectively. Each of the three morbidities was similarly and independently correlated with adverse 5-year outcome. The incidence of death or disability (95 % CI) in infants with none, any one, any two, and all three morbidities was 11.2 % (9.0 %–13.7 %), 22.9 % (19.6 %–26.5 %), 43.9 % (35.5 %–52.6 %), and 61.5 % (40.6 %–79.8 %), respectively. Among EPIs born at United State academic centers, modest reductions in several morbidities were observed, but BPD was increased over the last 20 years [23]. So how to reduce the high rates of in-hospital morbidity among EPIs who are provided ongoing intensive care remains a challenge for our neonatologists.

Multiple factors were associated with neonatal mortality and morbidity. In current study, it was identified that perinatal factors including antenatal steroids, gender, SGA, low Apgar score at 5 min, HDCP, placental abruption, placenta previa and multiple birth have an impact on the survival rate or morbidity rate. Previous study showed antenatal steroids use can improve the perinatal health in preterm infants [24, 25]. Our data showed the proportion of antenatal steroids was very low compared with other country [3, 11]. A possible explanation for the low incidence of antenatal steroids use in current study is the Chinese policy about “one child in one family” and futile medical care; the pregnancy will be terminated when the infants long-term outcomes are not as good as expected, especially in GA < 26 weeks [13–15]. ACOG Committee on Obstetric Practice recommended that a single course of corticosteroids should be administrated for pregnant women from 24 weeks to 34 weeks of GA who are at risk of preterm delivery within 7 days [26]. The 2013 European consensus guidelines for RDS have given the same recommendation on antenatal steroids use [27]. But in China, the present guideline from Obstetrics and Gynecology Society of Chinese Medical Association only suggested to give corticosteroids for pregnant women between 28 and 34 weeks of gestation, not including less than 28 weeks pregnant women [28]. Following the improving in neonatal intensive care, more and more EPIs were survived in China. So it is essential for our clinicians to start give active treatment strategy from 24 weeks.

The strengths of this study are the prospective, GA-based and a multicenter survey. The design of the study makes it was comparable to other GA-based cohort studies of extremely and very premature infants and provides information on the international position. Adopting methods from other countries in China might improve outcomes by analyzing multicenter data periodically for benchmarking, and assessing quality improvement initiatives. Our results may be valuable in counseling families and making novel interventions in China. We acknowledge inevitably there are several limitations. We only presented short-term outcomes in this study. The long-term neurodevelopmental outcome analysis is needed for this cohort study. The other disadvantage of this dataset is that the analysis is restricted to the information collected. We did not present any other data that might influence the outcomes of preterm infants such as maternal age, socio-economic status and details in clinical management strategy. Finally, this study was limited to liveborn preterm neonates of 24 to 31 complete weeks admitted to NICU, and we did not take into account any stillbirths and more immature babies less than 24 weeks. It hard to distinct if the cause of stillbirth at 24–25 weeks gestation was not influencing by the decisions of parents.

## Conclusion

In summary, we presented the hospital outcomes of extreme to very preterm infants from well-selective regional hospitals in China. Our study highlighted that many perinatal factors were associated with the mortality and morbidity besides gestational age and birth weight. Infants born at 24 and 25 weeks’ gestation were at higher risk of death than infants born at more than 26 weeks’ gestation. There is a need for further discussions on management for infants born at 24 and 25 weeks’ gestation.

## Abbreviations

BPD: Bronchopulmonary dysplasia
CI: Confidence interval
CNN: Canada neonatal network
EPI: Extremely preterm infants
GA: Gestational age
HDCP: Hypertensive disorder complicating pregnancy
IVH: Intraventricular hemorrhage
NEC: Necrotizing enterocolitis
NICU: Neonatal intensive care unit
NRNJ: Neonatal Research Network of Japan
PDA: Patent ductus arteriosus
PLA: the People’s Liberation Army
PROM: Premature rupture of membrane
RDS: Respiratory distress syndrome
ROP: Retinopathy of prematurity
SGA: Small for gestational age
VPI: Very preterm infants
